# Concentration-Response Relationships of the Non-enzymatic Scavenging Activity of Ethyl Pyruvate Against Multiple Free Radicals

**DOI:** 10.7759/cureus.99239

**Published:** 2025-12-14

**Authors:** Yomi Demura, Shigekiyo Matsumoto, Kazue Ogata, Kira Bacal, Takaaki Kitano, Osamu Tokumaru

**Affiliations:** 1 Faculty of Welfare and Health Sciences, Oita University, Oita, JPN; 2 Anesthesiology, Oita University, Oita, JPN; 3 Emergency Medicine, Faculty of Medical and Health Sciences, University of Auckland, Auckland, NZL; 4 Anesthesiology, Shin Beppu Hospital, Oita, JPN

**Keywords:** electron spin resonance, ethyl pyruvate, free radical species, oxidative stress, radical scavenging activity

## Abstract

Background

Pyruvate is a metabolic intermediate of energy metabolism that connects glycolysis and the tricarboxylic acid cycle. It also acts as an antioxidant, although it is unstable in solution. Ethyl pyruvate is a derivative of pyruvate with stability and lipid solubility and has been reported to have antioxidative activity. This study aimed to illustrate the concentration-response relationships of the non-enzymatic scavenging activity of ethyl pyruvate against multiple free radicals in vitro.

Methodology

Eight kinds of free radicals and singlet oxygen were generated in sample tubes. The direct scavenging activities of ethyl pyruvate against free radicals were evaluated by electron spin resonance spectroscopy using the spin-trapping method. Reaction rate constants were estimated from half-maximal inhibitory concentrations of concentration-response relationships.

Results

Ethyl pyruvate significantly scavenged the following six species of free radicals in concentration-dependent manners: hydroxyl radical (*k*_ethyl pyruvate_ = 1.4 × 10^8^ M^-1^s^-1^), superoxide anion (4.6 × 10^3^ M^-1^s^-1^), *tert*-butoxyl radical (0.91 × *k*_CYPMPO_), ascorbyl free radical (0.77 × *k*_edaravone_), singlet oxygen (0.19 × *k*_4-OH_TEMP_), and nitric oxide (8.6 M^-1^s^-1^). However, ethyl pyruvate did not scavenge *tert*-butyl peroxyl radical, *tert*-butyl hydroperoxide, 2,2-diphenyl-1-picrylhydrazyl, and tyrosyl radical.

Conclusions

It is speculated that the protective activity of ethyl pyruvate against oxidative stress might be attributable to non-enzymatic free radical scavenging activity.

## Introduction

Pyruvate (2-oxopropanoic acid) is a metabolic intermediate of energy metabolism. It connects the glycolysis and the tricarboxylic acid cycle as the final product of the former and the initial substrate for the latter. It also functions as a neuroprotective agent in vivo, acting as an antioxidant and free radical scavenger [1-3]. However, pyruvate is unstable in solution, making it difficult to use in clinical settings.

Ethyl pyruvate (ethyl 2-oxopropanoate) is a derivative of pyruvate with stability and lipid solubility. It also has antioxidative effects [4-7]. Our research group previously reported that ethyl pyruvate directly scavenges hydroxyl radicals [8]. Kładna et al. [9] reported its removal effect on reactive oxygen species by inhibiting chemiluminescent signals generated from superoxide anion in a dose-dependent manner. However, it has not yet been reported in detail whether ethyl pyruvate has direct (non-enzymatic) scavenging activity against other free radicals.

Therefore, we hypothesized that ethyl pyruvate would scavenge multiple kinds of free radicals, contributing to its antioxidative activity. The present study aimed to demonstrate the non-enzymatic scavenging activities of ethyl pyruvate against multiple free radical species and to illustrate its concentration-dependent properties.

## Materials and methods

Materials

We purchased ethyl pyruvate, *tert*-butyl hydroperoxide, and 2,2-diphenyl-1-picrylhydrazyl (DPPH) from Sigma-Aldrich (St. Louis, MO, USA). 5,5-dimethyl-1-pyrroline-*N*-oxide (DMPO), N-methyl-3-(1-methyl-2-hydroxy-2-nitrosohydrazino)-1-propanamine (NOC7), and 2-(4-carboxypheyl)-4,4,5,5-tetramethylimidazole-1-oxyl 3-oxide (carboxy-PTIO) were the products of Dojindo (Kumamoto, Japan). 5-(2,2-dimethyl-1,3-propoxy cyclophosphoryl)-5-methyl-1-pyrroline *N*-oxide (CYPMPO) was purchased from Mikuni Pharmaceutical Industrial (Osaka, Japan). Sodium ascorbate, dimethyl sulfoxide, and hydrogen peroxide were the products of Wako Pure Chemical Industries (Osaka, Japan). 2-2’-Azobis (2-amidinopropane) dihydrochloride (AAPH), Acid Red 94, and 4-hydroxy-2,2,6,6-tetramethylpiperidine (4-OH TEMP) were commercially available from Tokyo Chemical Industry (Tokyo, Japan). Ethyl pyruvate and other reagents were dissolved in ultrapure water prepared in our laboratory (ORGANO PX-0060μ-000, Tokyo, Japan). DPPH was dissolved in 100% ethanol.

Electron spin resonance spectrometry

Electron spin resonance (ESR) spectrometry was conducted as previously described [10-14]. Free radicals were detected using an X-band ESR spectrometer (JES-RE1X, JEOL, Tokyo, Japan). The data were analyzed offline with the operation software WIN-RAD ver.1.20b (Radical Research Inc, Tokyo, Japan). The ultraviolet (UV) or visible (VIS) light was produced by a 200 W medium-pressure mercury/xenon arc, and guided through a quartz light guide into the ESR sample cavity. Free radicals were produced in disposable glass micro-hematocrit capillary tubes (CS-HMT-502, Kimble Chase, Vineland, NJ, USA). The typical instrument settings were as follows: room temperature (23℃); frequency 9.45 GHz with 100-kHz modulation; modulation width, 0.1 mT; time constant, 0.1 s; center field, 335.8 mT; sweep width, 7.5 mT; sweep time, 1 min; microwave power, 4 mW.

**Figure 1:**
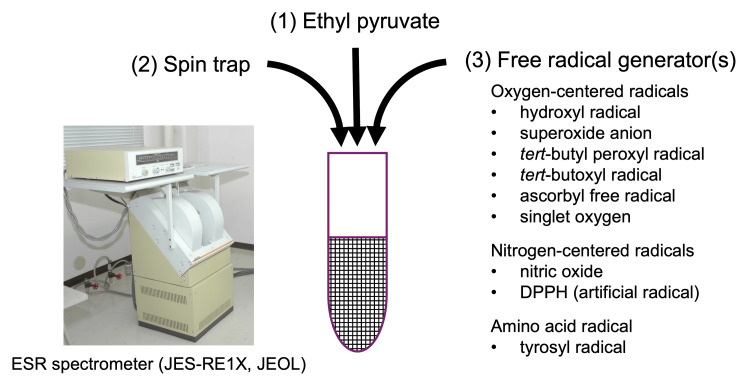
Experimental procedures. First, ethyl pyruvate (1) and a spin trap (2) were mixed in a test tube. Exactly 60 s after the addition of the free radical generator(s) (3), ESR measurement was started. ESR: electron spin resonance

Table 1 details the generation and trapping methods for the eight free radical species and singlet oxygen [10-14]. The reaction mixture (Figure 1) was composed of three parts: the subject drug (ethyl pyruvate), a spin-trap, and free radical generator(s). First, the spin-trap was added to the drug, followed by the addition of the free radical generator(s). Exactly 60 s after the free radical generator(s) was (were) added to the mixture, ESR measurement was started at each experiment. Briefly, hydroxyl radicals were generated by UV irradiation of hydrogen peroxide and trapped with CYPMPO. Superoxide anions were generated by the mixture of hypoxanthine and xanthine oxidase and trapped with DMPO. The *tert*-butyl peroxyl radical was generated by UV irradiation of *tert*-butyl hydroperoxide and trapped with CYPMPO. The *tert*-butoxyl radical was produced by UV irradiation of 3 mM AAPH and trapped with CYPMPO. Ascorbyl free radicals were studied by adding 99% dimethyl sulfoxide to sodium ascorbate [15]. Singlet oxygen was produced by VIS light irradiation of Acid Red 94 with its quencher, 4-OH TEMP. Nitric oxide was generated from NOC7 and reacted with carboxy-PTIO. DPPH was resolved in 100% ethanol. The tyrosyl radical was produced with hemoglobin and hydrogen peroxide and trapped with DMPO. Times from the generation of free radicals to the ESR measurement were strictly controlled so that they were kept constant (60 s) throughout the experiments.

**Table 1:** Generation of free radicals and reactive oxygen species. Final concentrations are given in the table. AAPH: 2-2’-Azobis (2-amidinopropane) dihydrochloride; carboxy-PTIO: 2-(4-carboxypheyl)-4,4,5,5-tetramethylimidazole-1-oxyl 3-oxide; CYPMPO: 5-(2,2-dimethyl-1,3-propoxy cyclophosphoryl)-5-methyl-1-pyrroline *N*-oxide; DMPO: 5,5-dimethyl-1-pyrroline-*N*-oxide; DMSO: dimethyl sulfoxide; DPPH: 2,2-diphenyl-1-picrylhydrazyl; DTPA: diethylenetriaminepentaaceticacid; NOC7: N-methyl-3-(1-methyl-2-hydroxy-2-nitrosohydrazino)-1-propanamine; 4-OH TEMP: 4-hydroxy-2,2,6,6-tetramethylpiperidine

Free radical species	Precursor/Sensitizer	Spin trap/Quencher
Hydroxyl radical	0.4% hydrogen peroxide + UV 4 s	0.33 mM CYPMPO
Superoxide anion	0.04 mM hypoxanthine + 0.02 U/mL xanthine oxidase	6.4 × 10^2^ mM DMPO
*tert*-Butyl peroxyl radical	80 mM *tert*-butyl hydroperoxide + 0.1 mM DTPA + UV 15 s	5.0 mM CYPMPO
*tert*-Butoxyl radical	1.4 mM AAPH + UV 4 s	3.8 mM CYPMPO
Ascorbyl free radical	0.26 mg/ml sodium ascorbate + 42% DMSO	―
Singlet oxygen	0.17 mM Acid Red + 500-600 nm light 60 s	1.7 mM 4-OH TEMP
Nitric oxide	140 μM NOC7	14 μM carboxy-PTIO
DPPH	15 μM DPPH	―
Tyrosyl radical	0.11 mM myoglobin + 0.002% hydrogen peroxide	6.0 × 10^2^ mM DMPO

Ratios of heights of ESR signals of target free radicals to those of Mn^2+^ were calculated. Then ratios were standardized by the control ESR signal with no ethyl pyruvate added.

Calculation of half-maximal inhibitory concentration

Data were nonparametrically fitted to the following Cheng-Prusoff’s sigmoid curve [16] to estimate concentration-response relationships.

$y = \frac{1}{1+(\frac{x}{a})^b}$

where *a* gives the estimation of half-maximal inhibitory concentration (IC_50_), *x* is the final concentration of ethyl pyruvate (M), and *y* is the observed free radical activity relative to the control. The Cheng-Prusoff’s sigmoid curve has been used by our laboratory in a series of studies on free radical scavenging activity of various drugs [10-14]. Despite its simple equation, it has successfully estimated the IC_50_ that always well matched the observed data.

Estimation of reaction rate constants

According to a kinetic competition model [17], the following competitive reactions occur in the reaction mixture:

spin trap + free radical $\rightarrow$ spin-adduct

ethyl pyruvate + free radical $\rightarrow$ ethyl-puruvate-radical

Given *k*_spin trap_ and *k*_ethyl pyruvate_ are the second-order rate constants of the respective reactions above, *k*_ethyl pyruvate_ is expressed as follows:

$k_{\text{ethyl pyruvate}}=\frac{\left[ \text{spin trap} \right]}{\mathrm{IC}_{50}}k_{\text{spin trap}}$

*k*_spin trap_ used were as follows: *k*_CYPMPO_ for hydroxyl radical 4.2 × 10^9^ M^-1^ s^-1^ [15], *k*_DMPO_ for superoxide anion 16 M^-1^ s^-1 ^[15], and *k*_cPTIO_ for nitric oxide 1.01 × 10^4^ M^-1^ s^-1^ [16]. Because *k*_CYPMPO_ for *tert*-butyl peroxyl radical and *tert*-butoxyl radical have not been reported, *k*_ethyl pyruvate_ for those free radicals was presented as relative values to *k*_CYPMPO_.

Statistical analysis

Statistical tests were conducted using the statistical software R ver.4.2.1 (https://www.R-project.org/). Values are presented as means (95% confidence intervals). The level of significance was 0.05.

## Results

**Figure 2:**
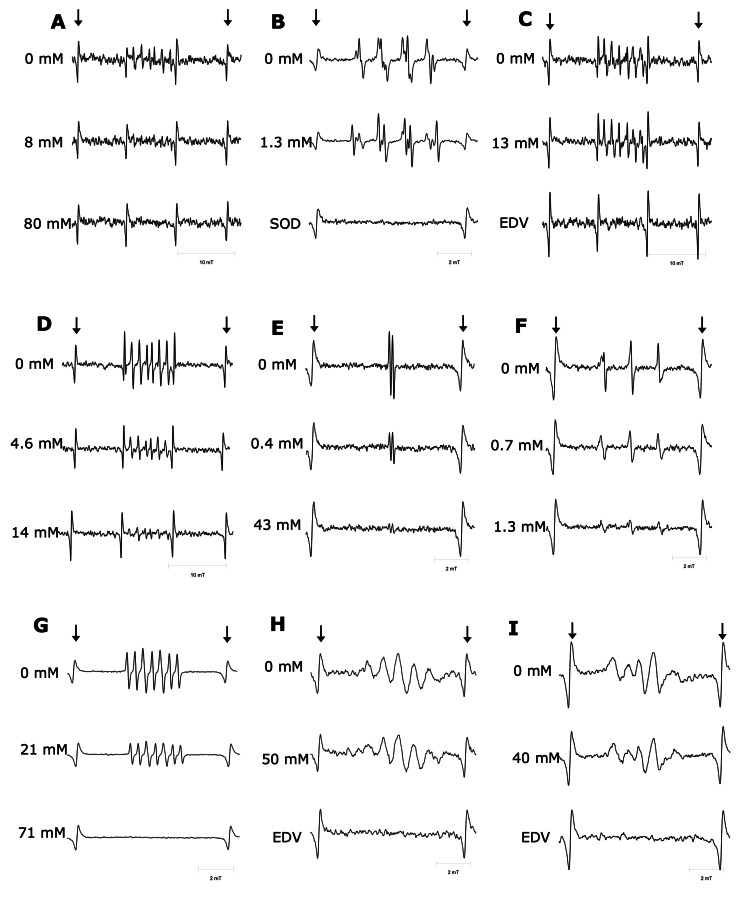
ESR spectra of free radicals. ESR spectra of hydroxyl radical (A), superoxide anion (B), *tert*-butyl peroxyl radical (C), *tert*-butoxyl radical (D), ascorbyl free radical (E), singlet oxygen (F), nitric oxide (G), DPPH (H), and tyrosyl radical (I). Final concentrations of ethyl pyruvate are shown on the left side of each panel. DPPH: 2,2-diphenyl-1-picrylhydrazyl; EDV: edaravone; ESR: electron spin resonance

The ESR spectra of the spin adducts for each radical examined are shown in Figure 2. Each spectrum was assigned to the corresponding free radical by the hyperfine splitting constants (Table 2).

**Table 2:** Half-maximal inhibitory concentration (IC50) and relative reaction rate constants against multiple free radicals and singlet oxygen. *k*_cPTIO_: reaction rate constant of 2-(4-carboxypheyl)-4,4,5,5-tetramethylomidazoline-1-oxyl 3-oxide; *k*_CYPMPO_: reaction rate constant of 5-(2,2-dimethyl-1,3-propoxy cyclophosphoryl)-5-methyl-1-pyrroline *N*-oxide; *k*_DMPO_: reaction rate constant of 5,5-dimethyl-1-pyrroline-*N*-oxide; *k*_EDV_: reaction rate constant of edaravone; *k*_4-OH_TEMP_: reaction rate constant of 4-hydroxy-2,2,6,6-tetramethylpiperidine

Free radical species	htfc (mT) a_H_, a_N_, a_P_	IC_50_ (mM)	Reaction rate constants		P-value
			relative value	absolute value (M^-1^s^-1^)	
Hydroxyl radical	1.37, 1.37, 4.88	9.8 ± 1.6	3.4×10^-2^ × k_CYPMPO_	1.4 × 10^8^	<0.01
Superoxide anion	1.15, 1.42, 0.13 (A_γH_)	2.2 ± 0.2	2.9×10^2^ × k_DMPO_	4.6 × 10^3^	<0.01
tert-Butyl peroxyl radical	1.35, 1.45, 5.05	―	―	―	―
tert-Butoxyl radical	1.24, 1.36, 4.80	4.2 ± 0.4	0.91 × k_CYPMPO_	―	<0.01
Ascorbyl free radical	0.186, ―, ―	0.22 ± 0.05	0.77 × k_Edv_	―	<0.01
Singlet oxygen	―, 1.50, ―	0.87 ± 0.06	0.19 × k_4-OH_TEMP_	―	<0.01
Nitric oxide	a_N1_ 0.981, a_N2_ 0.445	16 ± 2	8.6 × 10^-4^ × k_cPTIO_	8.6	<0.01
DPPH	―, 0.903, ―	―	―	―	―
Tyrosyl radical	―	―	―	―	―

Hydroxyl radical

**Figure 3:**
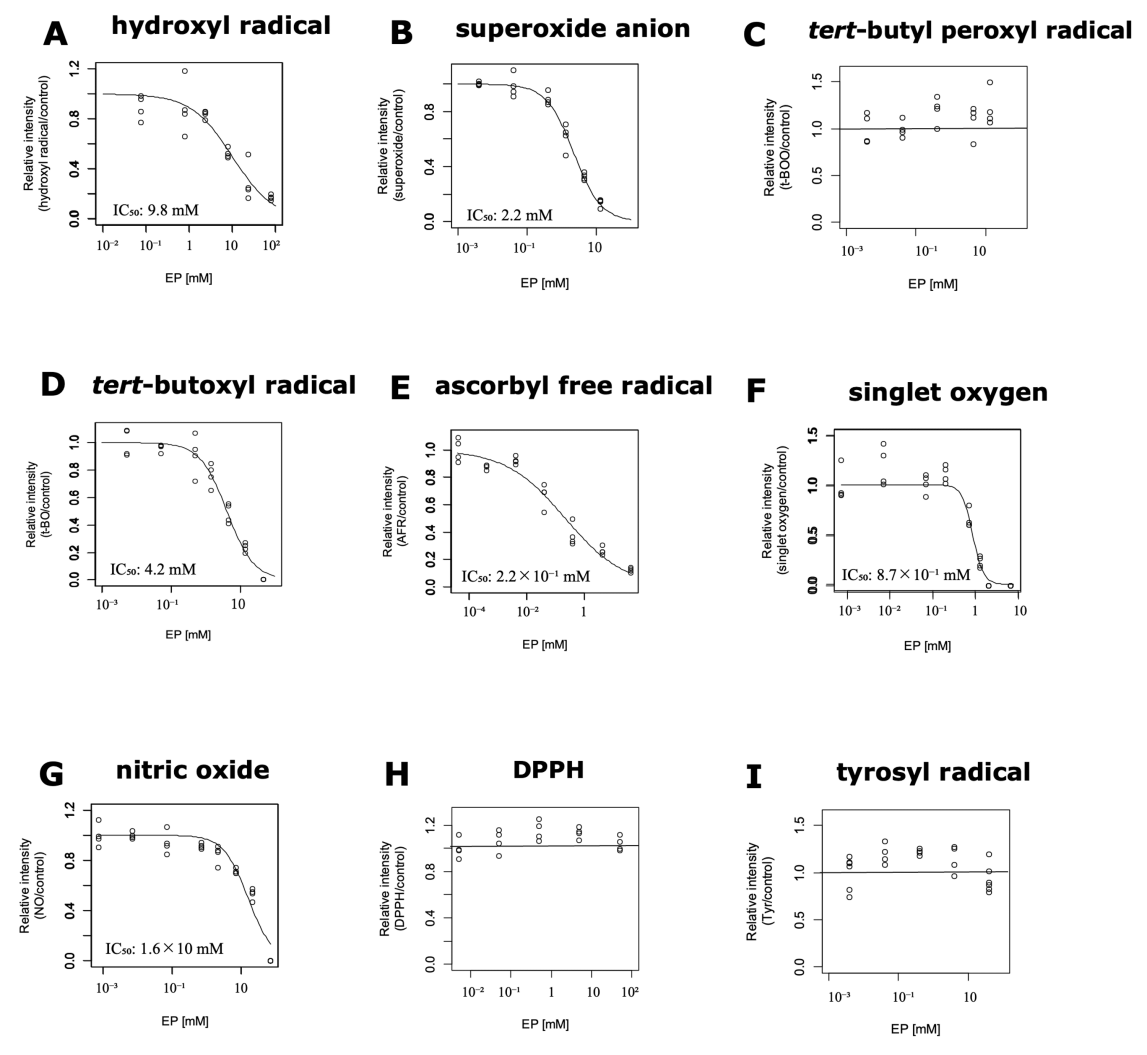
Concentration-response curves of non-enzymatic scavenging activity of ethyl pyruvate against free radicals and singlet oxygen. Ethyl pyruvate concentration-dependently scavenged hydroxyl radical (A), superoxide anion (B), *tert*-butoxyl radical (D), ascorbyl free radical (E), singlet oxygen (F), and nitric oxide (G). However, it did not show radical-scavenging activity against *tert*-butyl peroxyl radical (C), DPPH (H), and tyrosyl radical (I). Signals on both ends of each spectrum are those of the external standard of Mn^2+^. DPPH: 2,2-diphenyl-1-picrylhydrazyl

Ethyl pyruvate scavenged hydroxyl radicals as the concentration increased (Figure 2A). As shown in Figure 3A, ethyl pyruvate scavenged hydroxyl radicals in a concentration-dependent manner. By fitting the data to the Cheng-Prusoff’s equation [16], IC_50_ was determined to be 9.8 (95% confidence interval = 8.2-11.4) mM (p < 0.01). Using known *k*_CYPMPO_ = 4.2 × 10^9^ M^-1^s^-1^ for hydroxyl radical [18] and [CYPMPO] = 0.33 mM, *k*_ethyl pyruvate_ was estimated to be 1.4 × 10^8^ M^-1^s^-1^ (*k*_ethyl pyruvate_/*k*_CYPMPO_ = 3.4 × 10^-2^).

Superoxide anion

As shown in Figure 2B, ethyl pyruvate scavenged superoxide anion as the concentration increased. Superoxide anion was scavenged concentration-dependently by ethyl pyruvate (Figure 3B). By fitting the data to Cheng-Prusoff’s equation [16], IC_50_ was calculated to be 2.2 (2.0-2.4) mM (p < 0.01). Using *k*_DMPO_ = 16 M^-1^s^-1^ for superoxide anion [18] and [DMPO] = 6.4 × 10^2^ mM, *k*_ethyl pyruvate_ was estimated to be 4.6 × 10^3^ M^-1^s^-1^ (*k*_ethyl pyruvate_/*k*_DMPO_ = 2.9 × 10^2^).

*tert*-butyl peroxyl radical

Ethyl pyruvate did not scavenge *tert*-butyl peroxyl radicals at all (Figures 2C, 3C). As a positive control, 4.4 mM edaravone scavenged *tert*-butyl peroxyl radicals as previously reported [10] (Figure 2C).

*tert*-butoxyl radical

As the concentration of ethyl pyruvate increased, the ESR signal of *tert*-butoxyl radical reduced (Figure 2D). Ethyl pyruvate significantly scavenged *tert*-butoxyl radical in a concentration-dependent manner (Figure 3D). IC_50_ was calculated to be 4.2 (3.8-4.6) mM (p < 0.01). *k*_ethyl pyruvate_/*k*_CYPMPO_ was estimated to be 9.1 × 10^-1^. As the absolute value of *k*_CYPMPO_ has not been reported, *k*_ethyl pyruvate_ was presented as a relative value.

Ascorbyl free radical

Figure 2E shows that ethyl pyruvate scavenges ascorbyl free radicals as its concentration increases. Figure 3E illustrates the concentration-dependent scavenging activity of ethyl pyruvate against ascorbyl free radicals. Fitting the data to the Cheng-Prusoff’s equation demonstrated IC_50_ = 0.22 (0.17-0.27) mM (p < 0.01), which was equivalent to 0.77 × *k*_edaravone_ [10].

Singlet oxygen

Singlet oxygen was scavenged as the concentration of ethyl pyruvate increased (Figure 2F). By fitting the data plotted in Figure 3F to Cheng-Prusoff’s equation [10], IC_50_ was calculated to be 0.87 (0.81-0.93) mM (p < 0.01). *k*_ethyl pyruvate_/*k*_4-OH TEMP_ was estimated to be 0.19.

Nitric oxide

Ethyl pyruvate scavenged nitric oxide in a concentration-dependent manner (Figure 2G). By fitting the data plotted in Figure 3G to Cheng-Prusoff’s equation [10], IC_50_ was calculated to be 16 (14-18) mM (p < 0.01). Using *k*_cPTIO_ for nitric oxide of 1.01 × 10^4^ M^-1^ s^-1^ [19] and [cPTIO] = 0.014 mM, *k*_ethyl pyruvate_ was estimated to be 8.6 M^-1^s^-1^ (*k*_ethyl pyruvate_/*k*_cPTIO_ = 8.6 × 10^-4^).

DPPH

Ethyl pyruvate did not scavenge DPPH, an artificial free radical, at all (Figures 2H, 3H). As a positive control, 5 mM edaravone scavenged DPPH as previously reported [10] (Figure 2H).

Tyrosyl radical

Ethyl pyruvate did not scavenge tyrosyl radicals at all (Figures 2I, 3I). 4 mM Edaravone, a positive control, scavenged tyrosyl radical [11] (Figure 2I).

## Discussion

In the present study, there were only three players in a test tube: free radicals, spin traps, and ethyl pyruvate; the observed reaction occurred without enzymes. Thus, it was demonstrated that ethyl pyruvate non-enzymatically scavenged five kinds of free radicals and singlet oxygen out of the nine investigated in a non-enzymatic concentration-dependent manner.

Ethyl pyruvate, a stable and low-toxic derivative of pyruvate, has various pharmacological activities, including anti-inflammation, antioxidative stress, antiapoptosis, and antifibrosis [7-9,20]. As for anti-inflammatory effects, while it reduced the activation of nuclear factor kappa-B expression, ethyl pyruvate potentiated nuclear factor erythroid 2-associated factor 2 and downstream antioxidative molecules [21-23]. Ethyl pyruvate decreased the inflammatory responses by improving the T-helper 17/regulatory T cells imbalance [24]. Ethyl pyruvate also promoted growth arrest-specific gene 6/Axl signaling activation, which contributes to the immune restoration after the intracerebral hemorrhage mouse model, attenuating brain injury and enhancing neuroprotective effect [25].

Concerning antioxidative effects, Kładna et al. reported that ethyl pyruvate scavenged free radicals, including hydroxyl radical, superoxide anion, and nitric oxide [9]. However, they investigated the activity of ethyl pyruvate on the generation of reactive oxygen species by chemiluminescent signals, but not on its scavenging activity against free radicals [9]. Thus, it is still unclear which free radical species ethyl pyruvate directly scavenges.

Tokumaru et al. demonstrated that ethyl pyruvate was neuroprotective from the standpoint of energy metabolism; the recovery of phosphocreatine after ischemia/reperfusion was significantly improved when brain slices were superfused with artificial cerebrospinal fluid with ethyl pyruvate [8]. Intracellular pH was less acidic with ethyl pyruvate. They also reported that ethyl pyruvate scavenged hydroxyl radicals. However, concentration-dependency was not clearly illustrated; neither IC_50_ nor reaction rate constant was estimated at that time. In the present study, the concentration-dependency was illustrated for multiple species of free radicals, and the reaction rate constants were estimated for the first time. Ethyl pyruvate scavenges superoxide anion, which occurs at the most upstream stage of ischemia-reperfusion injury, and hydroxyl radical, which is one of the most harmful free radicals in vivo. Therefore, it would be expected that ethyl pyruvate could be used to suppress the oxidative stress and cell damage that occur downstream of these chain reactions.

It should also be noted that, compared with edaravone, ethyl pyruvate showed weaker scavenging activity against nitric oxide, which has a number of physiological effects in vivo, including dilation of vessels. This might indicate a possible benefit; i.e., the use of ethyl pyruvate might not interfere with the physiological effects of nitric oxide in vivo.

Ascorbate is a water-soluble antioxidant and is located at the most downstream of the oxidative chain reactions [26]. Unpaired electrons of free radicals produced intracellularly are transferred extracellularly via vitamin E in the cell membrane. Ascorbate acts as a donor of single reducing equivalents cycling between ascorbate and ascorbyl free radical, which makes ascorbate itself a free radical scavenger. Because ascorbyl free radical reacts preferentially with other radicals, making it not only a free radical scavenger but also a terminator of free radical chain reactions [27]. Thus, ascorbyl free radical is considered the final product of the chain reaction of oxidative stress and free radicals. Ethyl pyruvate could act as an antioxidant by scavenging not only the most upstream (i.e., superoxide anion) but also the most downstream products (i.e., ascorbyl free radical) of the chain reaction, with equivalent reaction rates to those of edaravone [10].

Although singlet oxygen is not a free radical but a reactive oxygen species, the scavenging activity of ethyl pyruvate was investigated. Singlet oxygen is generated in the skin by irradiation of photosensitizers by UV light in sunlight. Humans have no enzyme to eliminate singlet oxygen in the way that superoxide dismutase eliminates superoxide anions. Carotenoids are one of the major antioxidants to eliminate singlet oxygen [28,29]. The present result of the concentration-dependent scavenging activity of ethyl pyruvate against singlet oxygen could suggest possible clinical applications, such as the prevention of membrane lipid peroxidation.

*tert*-butoxyl radical and *tert*-butyl peroxyl radical are experimental reagents used to simulate alkoxyl radical and peroxyl radical of the oxidation chain reaction of lipids, including low-density lipoprotein-cholesterol. Ethyl pyruvate scavenged *tert*-butoxyl radical, suggesting that ethyl pyruvate might be preventive of the oxidative chain reaction of lipids. Meanwhile, it did not scavenge *tert*-butyl peroxyl radical. These results would suggest that the antioxidative activity of ethyl pyruvate might be limited, compared with that of edaravone, which scavenges both lipid radicals concentration-dependently [10,11].

Tyrosyl radical is the initial step of the cyclooxygenase reaction [30]. As ethyl pyruvate did not scavenge tyrosyl radical, its antioxidative and anti-inflammatory activities might be limited compared to edaravone, which directly scavenges tyrosyl radical [11].

The present study has some limitations. First, the possible influence of intracellular and intramitochondrial electrolytes, proteins, and other metabolites on scavenging activity was not considered. Second, the authors were only able to obtain absolute reaction rate constants for some of the free radicals examined. Third, the study was conducted only in vitro, and no in vivo experiments were included; there have been many in vivo studies reported supporting the antioxidative and anti-inflammatory activity of ethyl pyruvate. For the same reason, no interference with mitochondria was discussed where reactive oxygen species are generated in vivo. Future research must be conducted under conditions close to those of a living body.

## Conclusions

Ethyl pyruvate is a stable derivative of a metabolic intermediate of energy metabolism, which is reported to have antioxidative activities. In addition to its action through intracellular enzymatic pathways, we have demonstrated its direct non-enzymatic scavenging activity of ethyl pyruvate against specific species of free radicals, including hydroxyl radical and superoxide anion. It is speculated that some part of the antioxidative activity of ethyl pyruvate might be attributable to its direct non-enzymatic free radical scavenging activity. It would be possible that ethyl pyruvate could be clinically applied to prevent, for example, perioperative complications attributable to oxidative stress, such as perioperative arrhythmia and postoperative cognitive dysfunction. Ethyl pyruvate could protect patients from oxidative stress through not only enzymatic but also non-enzymatic pathways, as well as supplementing energy substrate.
